# Therapeutic Potential of Dental Pulp Stem Cell Secretome for Alzheimer's Disease Treatment: An In Vitro Study

**DOI:** 10.1155/2016/8102478

**Published:** 2016-06-14

**Authors:** Nermeen El-Moataz Bellah Ahmed, Masashi Murakami, Yujiro Hirose, Misako Nakashima

**Affiliations:** ^1^Department of Stem Cell Biology and Regenerative Medicine, National Center for Geriatrics and Gerontology, Research Institute, Obu, Aichi 474-8511, Japan; ^2^Department of Orodental Genetics, Division of Human Genetics and Human Genome, National Research Center, Cairo 12311, Egypt; ^3^Department of Oral and Molecular Microbiology, Osaka University Graduate School of Dentistry, Osaka 565-0871, Japan

## Abstract

The secretome obtained from stem cell cultures contains an array of neurotrophic factors and cytokines that might have the potential to treat neurodegenerative conditions. Alzheimer's disease (AD) is one of the most common human late onset and sporadic neurodegenerative disorders. Here, we investigated the therapeutic potential of secretome derived from dental pulp stem cells (DPSCs) to reduce cytotoxicity and apoptosis caused by amyloid beta (A*β*) peptide. We determined whether DPSCs can secrete the A*β*-degrading enzyme, neprilysin (NEP), and evaluated the effects of NEP expression in vitro by quantitating A*β*-degrading activity. The results showed that DPSC secretome contains higher concentrations of VEGF, Fractalkine, RANTES, MCP-1, and GM-CSF compared to those of bone marrow and adipose stem cells. Moreover, treatment with DPSC secretome significantly decreased the cytotoxicity of A*β* peptide by increasing cell viability compared to nontreated cells. In addition, DPSC secretome stimulated the endogenous survival factor Bcl-2 and decreased the apoptotic regulator Bax. Furthermore, neprilysin enzyme was detected in DPSC secretome and succeeded in degrading A*β*_1–42_ in vitro in 12 hours. In conclusion, our study demonstrates that DPSCs may serve as a promising source for secretome-based treatment of Alzheimer's disease.

## 1. Introduction

Alzheimer's disease is one of the most common neurodegenerative diseases that leads to dementia and decline of intellectual function. The pathology of AD is characterized by an accumulation of misfolded proteins, inflammatory changes, and oxidative stresses which result in loss of synaptic contacts and neuronal cell death [1]. It is estimated that the number of AD patients may increase to over 100 million cases by 2050 [2]. Therefore, the identification of new drugs and therapies is urgently needed to either prevent or delay the onset, slow the progression, or improve the symptoms of AD.

One of the main misfolded proteins in AD is amyloid beta (A*β*) peptide. Intensive therapeutic efforts have been attempted to treat AD targeting A*β*, including decreasing its accumulation [3, 4] and inhibiting the inflammatory process caused by it [5]. These therapies intended to delay the onset or slow the progression of AD. Among those potential therapies is the use of mesenchymal stem cells (MSCs). The therapeutic role of MSCs is based on their restorative and protective features rather than replacement of neuronal cells. Recent studies showed that MSCs can activate microglia [6], induce A*β* clearance, increase autophagy [7], induce neurogenesis [8], and enhance the level of synaptic transmission key proteins in AD models [9]. Additionally, growing stem cells release in culture medium some biologically active substances and structures, such as cytokines, growth factors, enzymes, microvesicles/exosomes, and genetic material [10, 11]. A recent report showed that adipose stem cells (ADSCs) can secrete functional neprilysin bound exosomes [12]. It is known that neprilysin (NEP) is a membrane-bound protease with efficient A*β* degradation activity. The levels of A*β* inversely correlate with the gene dosage of NEP and thus with its enzymatic activity [13]. In theory, the cultured stem cell secretome could be great pharmaceutical/medicinal product. Compared to cells, secretome could be easily biopreserved, sterilized, packaged, and stored. In context, the secretome obtained from mesenchymal stem cell cultures contains an array of neurotrophic factors and cytokines indicating the potential role in treating neurodegenerative conditions [14].

Dental pulp stem cells (DPSCs) are a unique type of mesenchymal stem cells. Besides their neural crest origin, DPSCs express pluripotent stem cell markers such as Oct4, Nanog, Sox, and Klf4 [15], and they have more potent neurogenicity and more immunosuppressive activities than bone marrow stem cells (BMSCs) [16]. All these properties actually make them better candidates for the treatment of neurodegenerative diseases. It has been reported that DPSCs are capable of stimulating long-term regeneration of nerves in the damaged spinal cord [17]. DPSCs promoted the regeneration of transected axons in a severed rat spinal cord by preventing multiple axon growth inhibitors and by preventing the apoptosis of neurons, astrocytes, and oligodendrocytes [18]. Moreover, DPSCs attenuated A*β*_1–42_ toxicity and increased the neuronal viability when cocultured with primary hippocampal neurons suggesting a neurotrophic effect [19].

To our knowledge, the therapeutic potential of DPSC secretome for AD has not been evaluated. In the present study, we characterized the DPSC secretome and investigated its neuroprotective effects against amyloid beta (A*β*) induced neurotoxicity in vitro. We examined the levels of anti- and proapoptotic factors, Bcl-2 and Bax, respectively, to investigate the role of DPSC secretome against A*β* induced apoptosis. Bcl-2 is one of the most important antiapoptotic factors that have a major role in stimulating the survival of cells while Bax is a proapoptotic factor that induces apoptosis and cell death. We also checked whether DPSCs can secrete the A*β*-degrading enzyme, neprilysin (NEP), and the effects of DPSC secretome in vitro by quantifying A*β*-degrading activity.

## 2. Methods 

### 2.1. Isolation and Culture of Dental Pulp Stem Cells

Normal human third molar teeth indicated for extraction were collected from patients aged 20–28 years at the Aichi-Gakuin University Dental Hospital, in accordance with the approved guidelines set by the School of Dentistry, Aichi-Gakuin University, and the National Centre for Geriatrics and Gerontology Research Institute. All experimental protocols were approved by the National Centre for Geriatrics and Gerontology Research Institute. Informed consent was obtained from all subjects involved in this experiment and donor information for used dental pulp derived mesenchymal stem cells can be found in Supplementary Table S1 in Supplementary Material available online at http://dx.doi.org/10.1155/2016/8102478. The pulp was gently removed using a sterile dental probe and the collected pulp tissue was dissected and digested in 0.2% Liberase MNP-S enzyme (Roche, Germany). The isolated dental pulp cells were cultured in Dulbecco Modified Eagle's Medium (DMEM) supplemented with 10% human serum and Antibiotic-Antimitotic solution (Gibco, Life Technologies) containing 500 units/mL of penicillin, 500 *μ*g/mL of streptomycin, and 1.25 *μ*g/mL of Fungizone^®^. Cells were selected on the basis of their ability to adhere to the dish; nonadherent cells were removed during medium replacement after 4-5 days in culture. Subsequent experiments have been performed in triplets on 3 different samples.

### 2.2. Preparation and Characterization of DPSC Secretome Using MAGPIX Cytokine Multiplex

For preparation of DPSC, BMSC, and ADSC secretomes (donor information for used bone marrow and adipose-derived mesenchymal stem cells can be found in Supplementary Tables S2 and S3), cells at passages (4th-5th) were grown to 60% confluency; then culture medium was switched to DMEM without serum and cells were starved for 24 hours. The medium was then collected and concentrated approximately 40-fold by Amicon Ultra-15 centrifugal filter unit with an ultracel-3 membrane (Millipore, Billerica, MA). Halt protease inhibitor cocktail (Thermo Scientific, USA) was added to the collected secretome at a concentration of 10 *μ*L/mL. Protein concentration was measured by Coomassie (Bradford) protein assay kit (Thermo Scientific, USA). The collected secretome was either used immediately or frozen at −30°C up to one month. Content analysis of DPSC secretome against BMSC and ADSC secretomes for cytokines, chemokines, and growth factors was performed using commercially available MAGPIX cytokine human 41 multiplex (Millipore) according to manufacturer's instructions. All data were compensated to pg/mL/10^6^ cells.

### 2.3. Preparation of A*β*_1–42_ Peptide

A*β*_1–42_ (Peptide Institute, Inc., Osaka, Japan) was provided as an amorphous powder that has been lyophilized from dimethyl sulfoxide (DMSO) solution. 1 mM solution was prepared by dissolving A*β*_1–42_ powder thoroughly in DMSO according to the manufacturer's instructions. The dissolved A*β*_1–42_ was used immediately after preparation.

### 2.4. SH-SY5Y Cells Viability Analysis and Morphological Assessment

Human neuroblastoma SH-SY5Y cells (Sanyo Chemical Industries, Ltd.) were cultured in DMEM/ham F12 (Sigma) supplemented with 10% FBS and Antibiotic-Antimitotic solution (Gibco, Life Technologies). In order to decide concentration and incubation time, SH-SY5Y cells were grown in 96-well plates for 24 h and then treated with A*β*_1–42_ (Peptide Institute, Inc., Osaka, Japan) over a range of concentrations (0 [control], 2, 5, or 10 *μ*M) for variable incubation times (0, 12, and 24 hours). The extent of cell growth and cell viability was assessed using a cell counting kit 8 assay (Dojindo Laboratories, Kumamoto, Japan) at 0, 12, and 24 h. Before assessment, the cells were washed and then 10 *μ*L of CCK-8 solution was added to each well, followed by incubation for 2 h at 37°C. The absorbance at 450 nm was determined by a multiplate reader (Thermo Scientific, Appliskan Multimode). Mean values of the mean absorbance rates from four wells were calculated. The neuroprotective effect of DPSC secretome was then tested in SH-SY5Y cells treated with 5 *μ*M of A*β*. SH-SY5Y cells were cultured for 24 hours; then the cells were divided into three groups exposed to either (i) 5 *μ*M of A*β*_1–42_, (ii) A*β*_1–42_ in combination with DPSC secretome (5 *μ*g/mL), or (iii) nonexposed cells as negative control. Cells were incubated for 24 hours in a humidified incubator at 37°C and 5% CO_2_. SH-SY5Y cells were then collected for assay. The viability of cells was assessed as previously described and morphological changes were observed under an inverted light microscope (Leica, 6000B-4) using Suite V3 (Leica).

### 2.5. Antiapoptotic Effect of DPSC Secretome

Cells were harvested from the three experimental groups and homogenized in 1X sample SDS lysis buffer. The protein concentration of cell lysates was determined by BCA protein assay (Thermo Scientific, Rockford, IL, USA) with bovine serum albumin as a standard. Ten micrograms of total protein was separated by electrophoresis in TGX*™* acrylamide gel (Bio-Rad) under reducing conditions and then electrophoretically transferred onto polyvinylidene fluoride (PVDF) membranes (immobilon-p, Millipore, USA). After protein transfer, the membranes were treated with 5% skim milk as a blocking buffer. The membranes were then probed with antibodies against either Bax (mouse, 610982, 1.5 : 1000, BD Transduction Laboratories, USA), Bcl-2 (mouse, 610538, 1.5 : 1000, BD Transduction Laboratories, USA), or *β*-actin (rabbit, RB-9421, 1 : 1000, Thermo Scientific, UK). Proteins of interest were detected with HRP-conjugated secondary antibody (1 : 1000, GE Healthcare, Uppsala, Sweden) and visualized with Luminata Forte Western HRP blotting substrate (Millipore, Germany) according to the provided protocol using light capture II cooled CCD camera system (ATTO cooperation, Japan). Detected bands were analyzed using Image J software (version 1.49r15).

### 2.6. Detection of Neprilysin/CD10 in DPSC Secretome

The protein level of neprilysin/CD10 in DPSC secretome was investigated and compared to its level in BMSC and ADSC secretomes using western blot analysis. After measuring the total protein concentration of secretomes using Coomassie (Bradford) protein assay, five micrograms of total protein was separated by electrophoresis under reducing conditions. PVDF membranes were incubated after blotting and blocking with primary antibody against neprilysin/CD10 (mouse, ab951, 1 : 1000, Abcam, USA). The membranes were visualized after secondary antibody treatment to detect and compare protein signals. Detected bands were analyzed using Image J software (version 1.49r15).

### 2.7. Degradation Ability of DPSC Secretome for A*β*_1–42_

Samples of 5 *μ*M A*β*_1–42_ were incubated at 37°C either with DPSC secretome (5 *μ*g/mL) or neprilysin/CD10 recombinant protein or alone for increasing length time (1, 3, 6, and 12 hours). At each time point, samples were collected and analyzed for remaining A*β* by western blot analysis using a primary antibody against A*β* (mouse, 10323, 1 : 1000, IBL, Japan).

### 2.8. Neuroprotective Ability of DPSC Secretome

SH-SY5Y cells were cultured for 10 days in serum-free DMEM-F12 to induce neurogenic differentiation. Cells were then divided into 3 groups: cells either exposed to A*β*_1–42_ only or A*β*_1–42_ and DPSC secretome or nonexposed cells. Undifferentiated SH-SY5Y cells were used as negative controls. Cells were incubated in a humidified incubator for 24 hours. Successful differentiation was confirmed using neuronal differentiation markers: neurofilament and neuromodulin (Supplementary Figure S6). Morphology and neurite lengths were then assessed. For neurite lengths assessment, multiple representative fields of cells morphology were photographed with an inverted light microscope (Leica, 6000B-4). Captured images were labeled with a scale according to the correspondent microscope magnification (×10). The images scale was used to convert pixels units into micrometers (*μ*m), using Image J software (version 1.49r15). The length of 5 to 10 neurites per field was traced and measured.

### 2.9. Statistical Analysis

Data are reported as mean ± standard error. Statistical analysis was performed using Microsoft (MS) Office Excel Software. One-way ANOVA was used to assess for differences between groups and *p* values were calculated using unpaired Student's *t*-test using IBM SPSS version 19. Differences were considered statistically significant if the *p* value was less than 0.05. The number of replicates in each experiment is indicated in the figure legends.

## 3. Results

### 3.1. Characterization of DPSC Secretome

DPSCs were successfully isolated from human third molar teeth indicated for extraction; their secretome was collected, analyzed, and compared to BMSC and ADSC secretomes using MAGPIX cytokine multiplex (Millipore). Various growth factors and cytokines were investigated as shown in Table 1.

**Figure 1:**
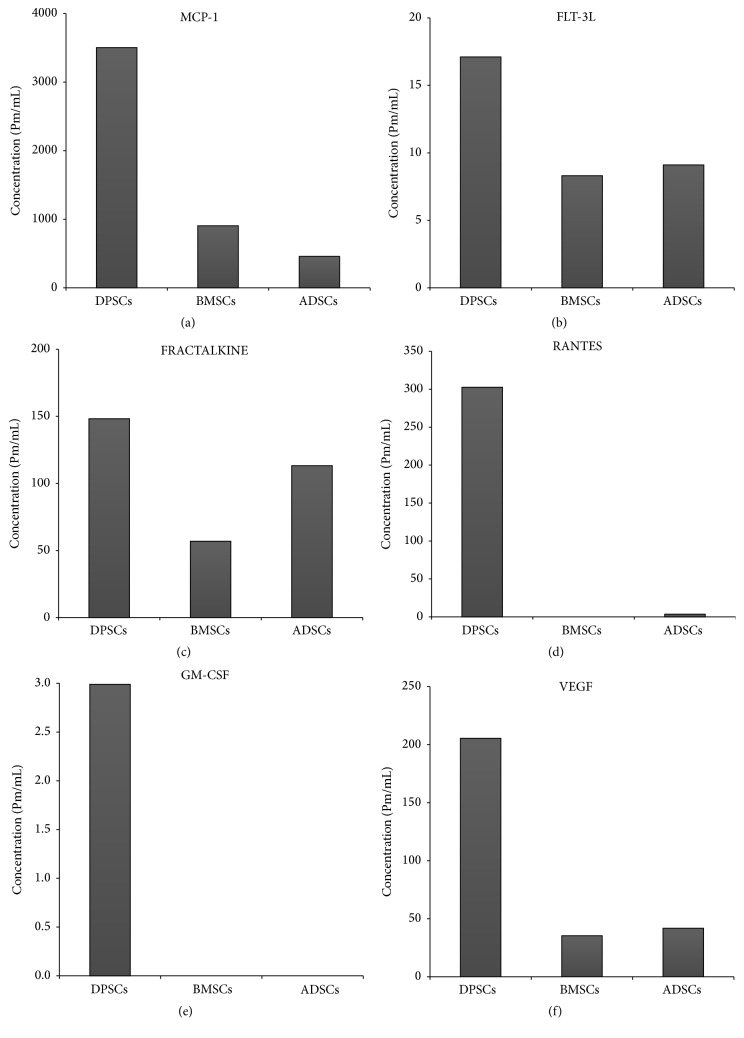
DPSCs secrete some cytokines and growth factors more than both BMSCs and ADSCs. (a) MCP-1. (b) FLT-3L. (c) FRACTALKIN. (d) RANTES. (e) GM-CSF. (f) VEGF.

Analysis showed that DPSC secretome contains higher concentrations of VEGF, RANTES, FRACTALKINE, FLT-3, GM-CSF, and MCP-1 than both BMSC and ADSC secretomes (Figure 1). Those factors and cytokines play important roles when it comes to neurodegenerative diseases. Some of these factors have neuroprotective effects like RANTES and VEGF [20, 21], while others may have antiapoptotic effects as MCP-1 [22] and FRACTALKINE [23]. FLT-3 can regulate microglial activation [24] and G-MCSF reverse cognitive impairment and amyloidosis [25]. These results indicate that DPSCs might have better therapeutic potentials for neurodegenerative diseases than other MSCs in terms of secreted neurotrophic factors.

### 3.2. Exposure to A*β* Reduces Cell Viability of SH-SY5Y Cells in a Dose- and Time-Dependent Manner

**Figure 2:**
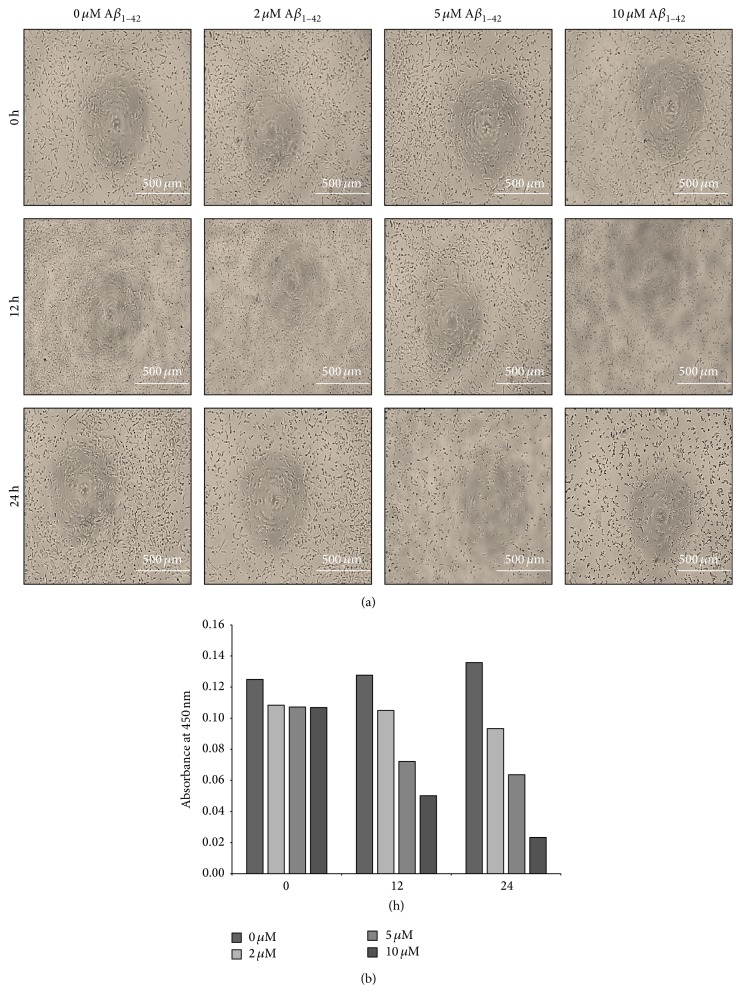
Exposure to A*β*_1–42_ decreased the viability of SH-SY5Y cells in a dose- and time-dependent manner. (a) Phase contrast micrographs of SH-SY5Y cells treated with various concentrations of A*β*_1–42_ for different periods of time. (b) Cell viability as detected at 450 nm absorbance (mean ± SE).

In order to determine the concentration and incubation time, neuroblastoma SH-SY5Y cells were treated with varying concentrations (0 [control], 2, 5, or 10 *μ*M) of A*β*_1–42_ (Peptide Institute, Inc., Osaka, Japan) for variable incubation times (0, 12, and 24 hours). A*β*_1–42_ treatment resulted in cellular morphological changes and loss of cellular viability in dose- and time-dependent manner (Figures 2(a) and 2(b)). The effect of A*β*_1–42_ on the viability of SH-SY5Y cells was most obvious after 24 hours and at 5 *μ*M concentration as the cytotoxic effect of A*β*_1–42_ was observed clearly without losing all cells. Thus, treatment with 5 *μ*M concentration of A*β*_1–42_ for 24 hours was chosen for the subsequent experiments.

### 3.3. DPSC Secretome Treatment Preserves Morphology and Improves the Viability of SH-SY5Y Cells

**Figure 3:**
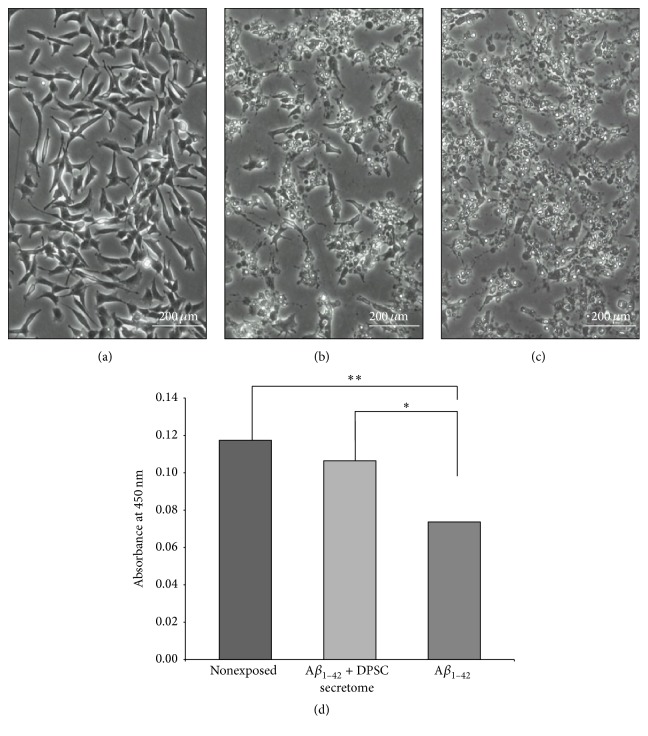
DPSC secretome treatment preserves morphology and improves the viability of SH-SY5Y cells exposed to A*β*_1–42_. (a–c) Phase contrast micrographs of SH-SY5Y cells exposed to A*β*_1–42_ only, A*β*_1–42_, and DPSC secretome or nonexposed as control (full-size images are presented in Supplementary Figure S1). (d) Cell viability as detected at 450 nm absorbance (mean ± SE, *n* = 3. ^*∗*^ *p* < 0.05, ^*∗∗*^ *p* < 0.01).

SH-SY5Y cells exposed to 5 *μ*M A*β*_1–42_ and treated with DPSC secretome (5 *μ*g/mL) were compared to nontreated cells for morphological changes. Cells treated with DPSC secretome appeared to preserve their intact morphological shape while obvious morphological changes have been noticed in nontreated cells (Figures 3(a), 3(b), and 3(c)). Moreover, there was a significant increased viability (*p* = 0.0103) in cells treated with DPSC secretome as total cell number was higher compared to nontreated cells (0.11 ± 0.008 and 0.07 ± 0.005, resp., *n* = 3, mean ± SE) (Figure 3(d)).

### 3.4. Antiapoptotic Effects of DPSC Secretome

**Figure 4:**
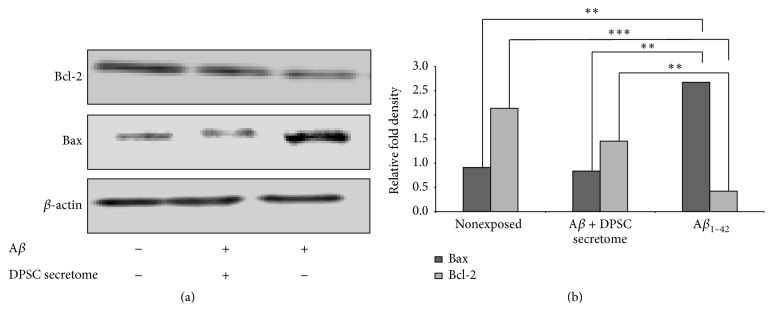
DPSC secretome stimulates the endogenous survival factor Bcl-2 and decreases the apoptotic regulator Bax. (a) Immunoblotting analysis with an anti-Bax, an anti-Bcl2, or an anti-actin antibody (full-length blots are presented in Supplementary Figure S2). (b) Relative fold density as analyzed by Image J (mean ± SE, *n* = 3. ^*∗∗*^ *p* < 0.01, ^*∗∗∗*^ *p* < 0.001).

SH-SY5Y cells were divided into three groups: cells either exposed to (i) 5 *μ*M of A*β*_1–42_ alone or (ii) A*β*_1–42_ in combination with DPSC secretome (5 *μ*g/mL) or (iii) Nonexposed cells as negative control. Cells lysates from the three groups were analyzed by western blotting to detect expression of Bcl2 (antiapoptotic factor) and Bax (apoptotic regulator). DPSC secretome treated cells showed a significant increase in Bcl2 expression (*p* = 0.000) compared to the nontreated cells (1.46 ± 0.025 and 0.42 ± 0.06, resp., *n* = 3, mean ± SE). On the other hand, Bax expression was significantly decreased (*p* = 0.001, 0.84 ± 0.03 and 2.6741 ± 0.18, *n* = 3, mean ± SE) (Figures 4(a) and 4(b)). These results indicate that DPSC secretome has an antiapoptotic effect by stimulating the endogenous survival factor Bcl-2 and decreasing the apoptotic regulator Bax, revealing the possible mechanism of neuroprotection.

### 3.5. Neprilysin (NEP) Content in DPSC Secretome and Its Degrading Ability

**Figure 5:**
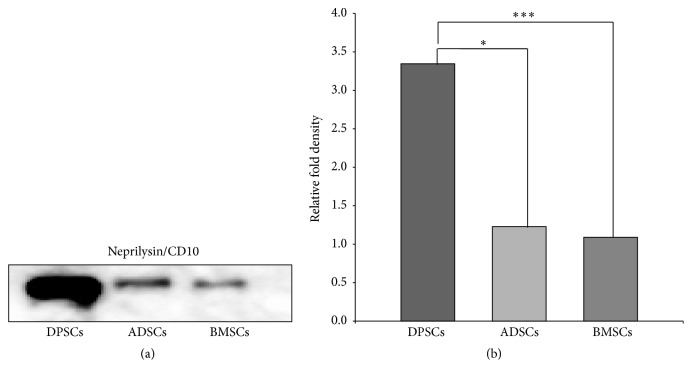
DPSC secretome contains a higher concentration of neprilysin/CD10. (a) Immunoblotting analysis with an anti-NEP antibody (full-length blots are presented in Supplementary Figure S3). (b) Relative fold density as analyzed by Image J (mean ± SE, *n* = 3. ^*∗*^ *p* < 0.05, ^*∗∗∗*^ *p* < 0.001).

**Figure 6:**
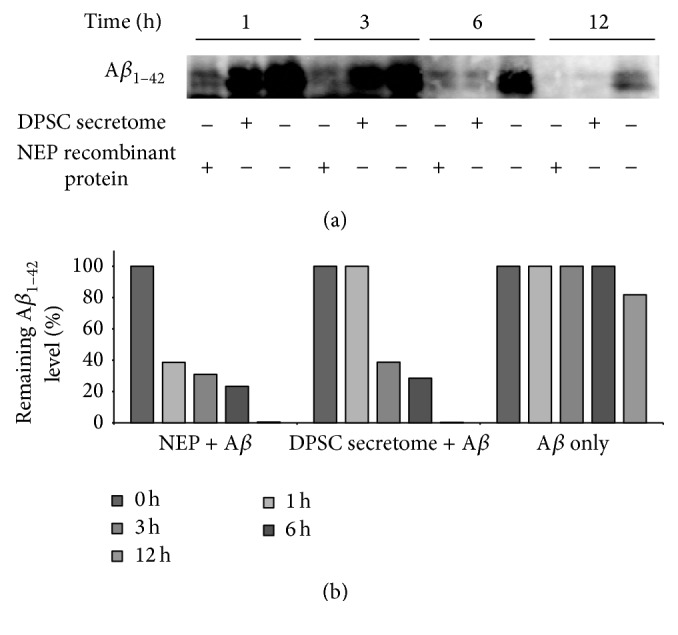
DPSC secretome degrades A*β*_1–42_ protein in vitro. (a) Immunoblotting analysis at each time point with an anti-A*β* antibody (full-length blots are presented in Supplementary Figure S4). (b) Quantitation of remaining A*β*_1–42_ level (mean ± SE).

NEP expression was significantly increased in DPSC secretome compared to that in both BMSC (*p* = 0.000) and ADSC (*p* = 0.01) secretomes (3.35 ± 0.073, 1.23 ± 0.16, and 1.09 ± 0.28, resp., *n* = 3, mean ± SE), indicating the stronger potential of DPSCs to degrade A*β*_1–42_ protein (Figures 5(a) and 5(b)). We sought to determine whether DPSC secretome could proteolytically degrade A*β*_1–42_ in vitro. Quantitative immunoblotting analysis showed that incubation with 5 *μ*g/mL of DPSC secretome resulted in complete degradation of 5 *μ*M of A*β*_1–42_ after 12 hours (Figure 6). However, the rate of A*β*_1–42_ hydrolysis was relatively slow at the first 3 hours when compared to hydrolysis by NEP recombinant protein.

### 3.6. Neuroprotective Ability of DPSC Secretome

**Figure 7:**
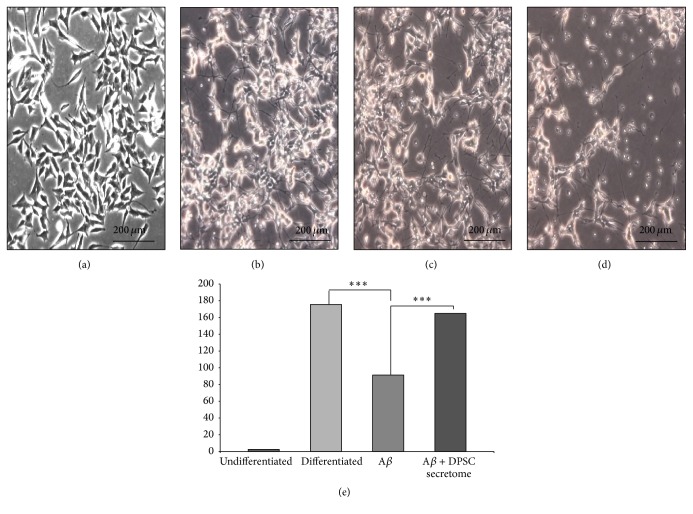
DPSC secretome has neuroprotective ability against A*β*_1–42_ induced neurotoxicity. (a–d) Representative photos demonstrating the morphology of SH-SY5Y cells in different treatment groups: undifferentiated, nonexposed differentiated, differentiated exposed to A*β*_1–42_ and DPSC secretome, and differentiated exposed to A*β*_1–42_ only (full-size images are presented in Supplementary Figure S5) (e). Quantitative analysis of SH-SY5Y neurite outgrowth after exposure to A*β*_1–42_ for 24 hours (mean ± SE, *n* = 3, ^*∗∗∗*^ *p* < 0.001).

Neurodifferentiated SH-SY5Y exposed to A*β*_1–42_ and treated with DPSC secretome kept their morphology and viability compared to nontreated cells. Cells not treated with DPSCs secretome showed decreased viability (Figures 7(a)–7(d)) and significant shrinkage in neural extensions compared to treated cells (165 ± 3.2 and 91 ± 3.6, resp., *n* = 3, *p* = 0.000, mean ± SE) (Figure 7(c)), indicating the neuroprotective ability of the DPSC secretome against A*β*_1–42_ induced neurotoxicity.

## 4. Discussion 

Recent studies on mesenchymal stem cells (MSCs) have provided promising new ways for tissue repair in central nervous system diseases [26]. Initially, it was considered that the true therapeutic potential of MSCs depends mainly on their ability to differentiate into multiple lineages, but recently it has been shown that their therapeutic potential was mostly related to the growth factors that they secrete rather than to their differentiation potential [27]. In the present study, we evaluated the therapeutic potential of DPSC secretome for AD treatment. Our results demonstrated that DPSC secretome was able to reduce the toxic effect of A*β*_1–42_ on neuroblastoma cells, secrete A*β*-degrading enzyme neprilysin, and decrease A*β*_1–42_ induced apoptosis. To our knowledge, this is the first report to investigate the therapeutic potentials of DPSC secretome for Alzheimer's disease.

During the organogenesis of the tooth, dental stem cells play critical roles by secreting neurotrophic factors such as NGF (nerve growth factor), GDNF (glial cell-derived neurotrophic factor), and BDNF (brain-derived neurotrophic factor) which results in innervation of dental tissues [28]. This neurotrophic feature brings an important advantage to dental stem cells in terms of restoration of neuronal tissues. In our study, we started by characterizing the secretome collected from DPSCs in an attempt to know its content and compare it to other MSC secretomes. We have identified that DPSCs secrete (in addition to the previously mentioned factors) other important growth factors and cytokines which might be involved in attenuation of neurotoxicity such as VEGF, RANTES, FRACTALKINE, FLT-3, and MCP-1. Furthermore, DPSCs were found to secrete these cytokines and factors at higher concentrations than both ADSCs and BMSCs. While VEGF secretion enhances angiogenesis during tissue repair [22], RANTES was found to increase neuronal cell survival and to have a neuroprotective effect [20], and FRACTALKINE is considered as a key microglial pathway in protecting against AD-related cognitive deficits [23]. Moreover, FLT-3 is involved in microglial cells' capacity to respond to environmental cues to function as antigen presenting cells and mediate CNS inflammation, suggesting that FLT-3 may be a therapeutic target on microglia to alleviate CNS inflammation [24]. Furthermore, G-MCSF can reverse cognitive impairment and amyloid pathology in AD mice [25]. Taken together, the data presented indicate that DPSC secretome has high therapeutic potentials in neurodegenerative diseases.

We evaluated the ability of the DPSC secretome to protect neuroblastoma cells (SH-SY5Y) against A*β*_1–42_ induced toxicity. The exposure of the SH-SY5Y cultures to A*β* led to a reproducible and dose-time-dependent loss in cell viability. This toxic effect was attenuated when the cells were cotreated with DPSC secretome. It has been previously demonstrated that coculturing with DPSCs significantly reduced A*β*_1–42_ induced toxicity in primary cultures of mesencephalic and hippocampal neurons and led to an increase in neuronal viability [19]. This can now be attributed to the factors that DPSCs secrete in culture.

Several approaches have been made to investigate the potential role of A*β* in the apoptotic mechanism of AD. Initial studies suggested that A*β* plaques directly induce apoptosis in vitro [29]. However, it was suggested that A*β* is more likely to induce apoptosis indirectly, possibly by first promoting oxidative stress through the production of reactive oxidative species (ROS) [30]. A*β* also activates apoptosis pathway by upregulating the proapoptotic Bax protein and inducing other apoptotic signal cascades [31]. In the present study, we showed that DPSC secretome upregulated Bcl-2 and downregulated* Bax* expression in SH-SY5Y cells counteracting the effect of A*β*. Bax and caspase-3 have been implicated in the pathogenesis of AD and are components of a well-defined molecular pathway of neuronal apoptosis [32]. And given that Bcl-2 inhibits Bax-induced apoptosis, delays caspase expression, and increases neuron survival [33], it will be expected that manipulating the expression and or the activity of Bcl-2 will increase neuronal survival. Growth factors and cytokines like FGF2 [34], VEGF [35], RANTES [36], and Fractalkine [37] can upregulate Bcl-2 expression. And the data presented here proves that those factors not only are found abundantly within the DPSC secretome but also are found at higher concentrations than in other MSC secretomes. Accordingly, DPSC secretome exhibits those antiapoptotic properties.

Recently, there has been considerable debate over the enzyme or enzymes that contribute most to A*β* degradation in human brain. It is likely that multiple proteases, both intracellular and extracellular, may play a role in determining A*β* concentration in human brain. Neprilysin (NEP) is one of the major proteases involved in that debate. NEP is a membrane-bound protease with efficient A*β* degradation activity. It has been found that levels and activity of NEP are decreased in AD brains, suggesting that a reduction in A*β* degradation may contribute to the development of the disease [38]. When it comes to NEP and MSCs, a study showed that the coculture of human MSCs and mouse microglia increased neprilysin expression under the exposure of A*β* [39]. It has been also reported that adipose stem cells (ADSCs) can secrete functional neprilysin bound exosomes [12]. In our study, we investigated the expression of neprilysin within the DPSC secretome at a protein level using western blot analysis. Our results showed that DPSC secretome contains functional NEP that was able to degrade 5 *μ*M of A*β*_1–42_ in vitro within 12 hours. Another important observation was that DPSCs expressed NEP at a higher level than both BMSCs and ADSCs. These results suggest that DPSC secretome has the capacity to contribute to clearance of A*β* accumulated in the brain, indicating that DPSCs may serve as a promising source for secretome-based AD treatment.

In conclusion, the results of the present study support the use of DPSCs as a source of secretome which is highly enriched in neurotrophic factors, A*β*-degrading enzyme (NEP), and antiapoptotic factors, rendering DPSCs promising candidates for secretome-based therapy for AD providing a novel therapeutic approach against one of the most common neurodegenerative diseases.

## Supplementary Material

Supplementary Table 1: Donor information for used dental pulp derived mesenchymal stem cells.
Supplementary Table 2: Donor information for used bone marrow-derived mesenchymal stem cells.
Supplementary Table 3: Donor information for used Adipose-derived mesenchymal stem cells.
Supplementary Figure 1: DPSC secretome treatment preserves morphology and improves viability of SH-SY5Y cells exposed to A*β*1–42.
Supplementary Figure 2: DPSC secretome stimulates the endogenous survival factor Bcl-2 and decreases the apoptotic regulator Bax.
Supplementary Figure 3: DPSC secretome contains higher concentration of Neprilysin/CD10.
Supplementary Figure 4: DPSC secretome degrade A*β*1–42 protein in vitro.
Supplementary Figure 5: DPSC secretome has neuroprotective ability against A*β*1–42 induced neurotoxicity.

## Figures and Tables

**Table 1:** List of cytokines and growth factors investigated by MAGPIX cytokine multiplex.

Name of cytokine/growth factor	Abbreviation/another name
Endothelial growth factor	EGF
Fibroblast growth factor-2	FGF-2
Fms-related tyrosine kinase 3	FLT-3L
FRACTALKINE	CX3CL1
Granulocyte-macrophage colony-stimulating factor	GM-CSF
Human platelet-derived growth factor BB	PDGF-BB
Interferon alpha 2 and gamma	INF*α*2, INF*γ*
Interleukin-12 p40	IL-12p40
Interleukin-12 p70	IL-12p70
Interleukin-13	IL-13
Interleukin-15	IL-15
Interleukin-17	IL-17
Interleukin-1 receptor antagonist	IL-1ra
Interleukin-1 alpha and beta	IL-1*α*, IL-1*β*
Interleukin (2–10)	IL-(2–10)
Macrophage-derived chemokine	MDC
Macrophage inflammatory protein 1 alpha and beta	MIP-1*α*, MIP-1*β*
Monocyte chemotactic protein 1 and 3	MCP-1, MCP-3
Platelet-derived growth factor-AA	PDGF-AA
RANTES	CCL5
CXCL10	IP-10
Soluble CD40 ligand	sCD40L
Tumor necrosis factor alpha and beta	TNF*α*, TNF*β*
Vascular endothelial growth factor	VEGF
