# Supplementary material for: Therapeutic Potential of Dental Pulp Stem Cell Secretome for Alzheimer's Disease Treatment: An In Vitro Study
Source: Stem Cells Int. 2016 Jun 14;2016:8102478. doi: 10.1155/2016/8102478 (PMC4923581; doi:10.1155/2016/8102478)
Supplement: Supplementary file 1 — Supplementary Table 1: Donor information for used dental pulp derived mesenchymal stem cells. Supplementary Table 2: Donor information for used bone marrow-derived mesenchymal stem cells. Supplementary Table 3: Donor information for used Adipose-derived mesenchymal stem cells. Supplementary Figure 1: DPSC secretome treatment preserves morphology and improves viability of SH-SY5Y cells exposed to Aβ1–42. Supplementary Figure 2: DPSC secretome stimulates the endogenous survival factor Bcl-2 and decreases the apoptotic regulator Bax. Supplementary Figure 3: DPSC secretome contains higher concentration of Neprilysin/CD10. Supplementary Figure 4: DPSC secretome degrade Aβ1–42 protein in vitro. Supplementary Figure 5: DPSC secretome has neuroprotective ability against Aβ1–42 induced neurotoxicity. [file 8102478.f1.docx]

**Supplementary information**

**Therapeutic potential of Dental pulp stem cell secretome for Alzheimer’s disease treatment: an in vitro study.**

Nermeen EL-Moataz Bellah Ahmed, Masashi Murakami, Yujiro Hirose & Misako Nakashima

- **Supplementary Table 1. Donor information for used dental pulp derived mesenchymal stem cells.**
- **Supplementary Table 2. Donor information for used bone marrow-derived mesenchymal stem cells.**
- **Supplementary Table 3. Donor information for used Adipose-derived mesenchymal stem cells.**
- **Supplementary figure legends :**

**Fig. S1. DPSC secretome treatment preserves morphology and improves viability of SH-SY5Y cells exposed to Aβ_1-42_.** Full size pictures shown of SH-SY5Y cells exposed to Aβ_1-42_ only, Aβ_1-42_ and DPSC secretome or non-exposed as control.

**Fig. S2. DPSC secretome stimulates the endogenous survival factor Bcl-2 and decreases the apoptotic regulator Bax.** Full length scans of western blot membranes treated with an anti-Bax, anti-Bcl2 or anti-actin antibody.

**Fig. S3. DPSC secretome contains higher concentration of Neprilysin/CD10.** Full-length scans of western blot membranes treated with anti-NEP antibody.

**Fig. S4. DPSC secretome degrade Aβ_1-42_ protein in vitro.** Full-length scans of western blot membranes treated with an anti-Aβ antibody.

**Fig. S5. DPSC secretome has neuroprotective ability against Aβ_1-42_ induced neurotoxicity.** Full size pictures of representative photos demonstrating the morphology of SH-SY5Y cells in different treatment groups; undifferentiated, non-exposed differentiated, differentiated exposed to Aβ_1-42_ and DPSC secretome and differentiated exposed to Aβ_1-42_ only.

| **Table S1. Donor information for used dental pulp derived mesenchymal stem cells.** | | | | |
| --- | --- | --- | --- | --- |
| **ID** | #1 | #2 | #3 | #4 |
| **Sex** | Female | Female | Female | Female |
| **Age** | 22 | 25 | 23 | 21 |
| **Tooth** | Upper right third molar | Upper left third molar | Lower left third molar | Lower right third molar |

| **Table S2. Donor information for used bone marrow-derived mesenchymal stem cells.** | | | | |
| --- | --- | --- | --- | --- |
| **ID** | #1 | #2 |  |  |
| **Bank name** | Health science research resources | Lonza Walkersville, Inc |  |  |
| **Cell name** | UE6E7-16 | PT-2501 |  |  |
| **Sex** | Female | Female |  |  |
| **Age** | 91 | 21-22 |  |  |
| **Site** | Posterior iliac crest of the pelvic bone | Posterior iliac crest of the pelvic bone |  |  |

| **Table S3. Donor information for used adipose-derived mesenchymal stem cells.** | | | | | | |  |
| --- | --- | --- | --- | --- | --- | --- | --- |
| **ID** | #1 | |  |  | |  |  |
| **Bank name** | Lonza Walkersvillen, Inc |  | | |  | |  |
| **Cell name** | PT-5006 | |  |  | |  |  |
| **Site** | lipoaspirates | |  |  | |  |  |

**Fig. S1**


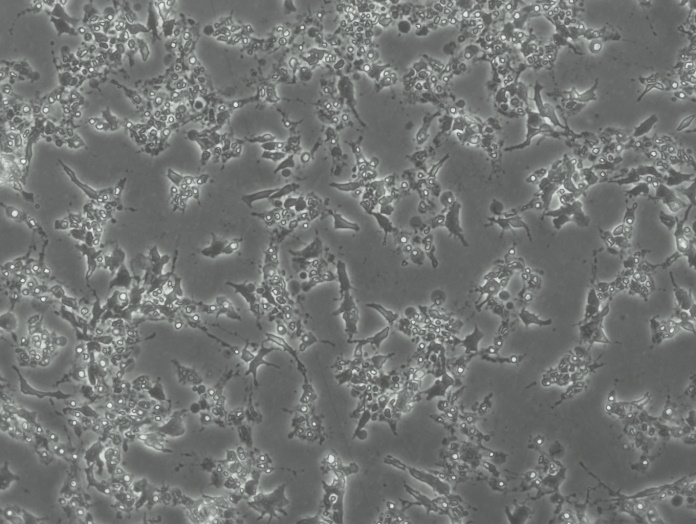

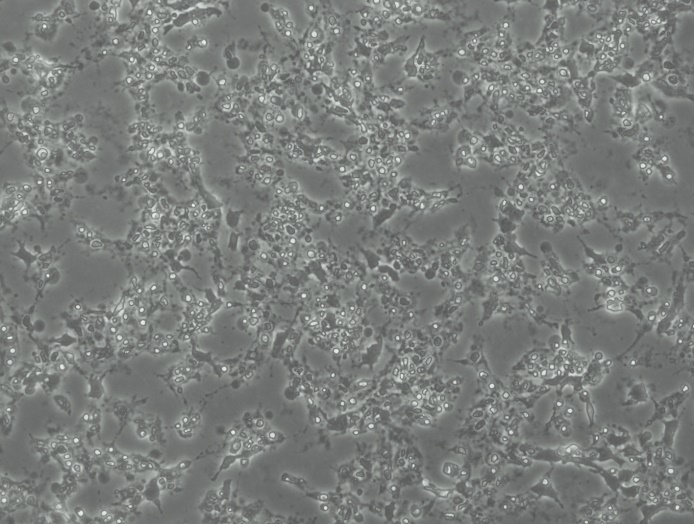

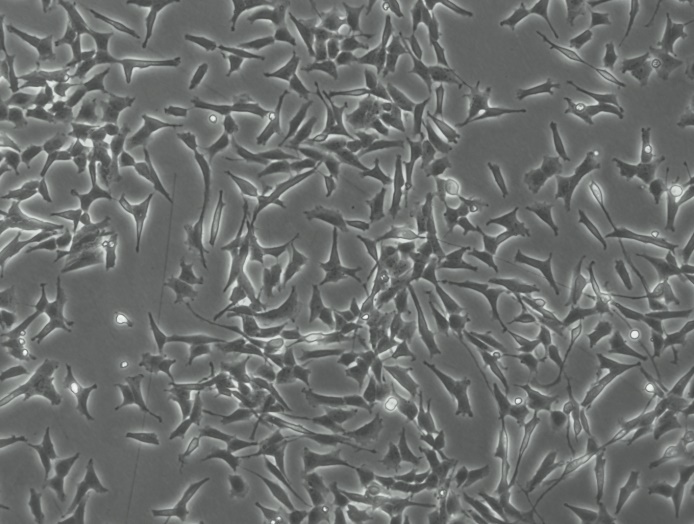


**Aβ only**

**Aβ and DPSC secretome**

**Non-exposed (control)**

**200µm**

**200µm**

**200µm**

**Fig. S2**


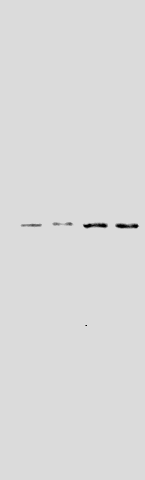

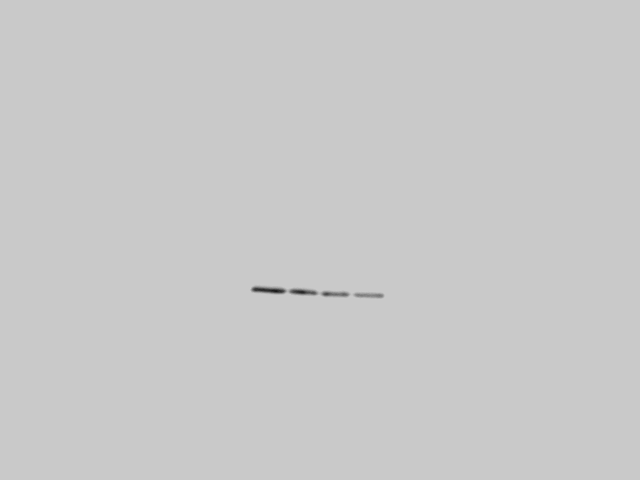

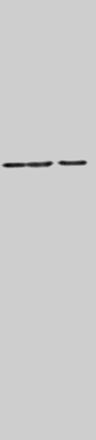


MW(kDa)

25 -

20 -

37 -

50 -

75 -

100 -

150 -

250 -

15 -

10 -

**Bcl-2**

**Bax**

**β-actin**

**Fig. S3**


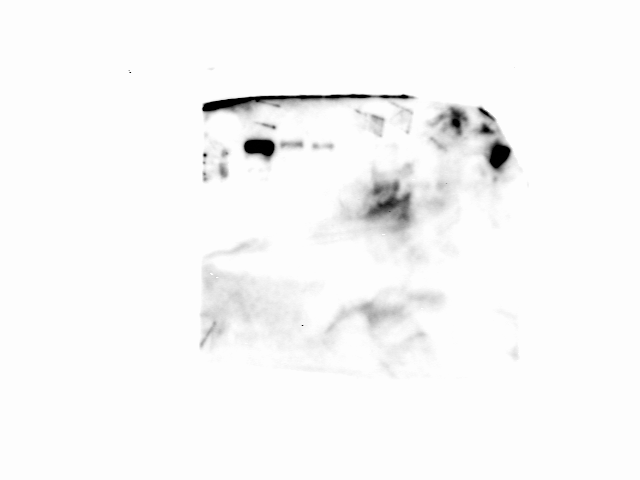


**100 -**

**MW(kDa)**

**Neprilysin/CD10**

**Fig. S4**


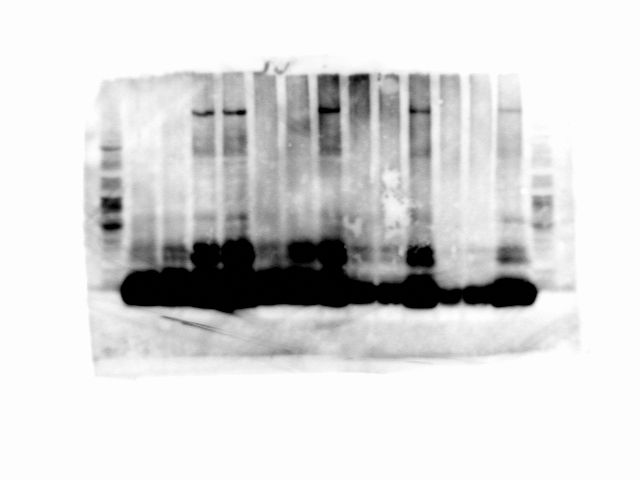


**MW(kDa)**

**10 kDa -**

**Fig. S5**

**Undifferentiated cells (-ve control)**

**Differentiated (Aβ+DPSC secretome)**

**Differentiated cells**


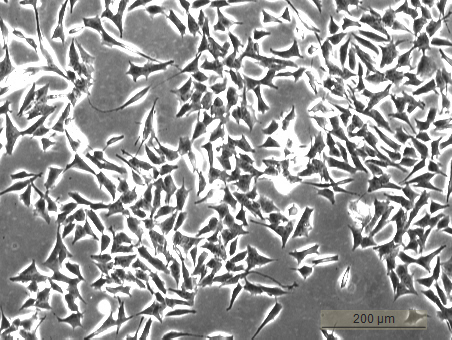

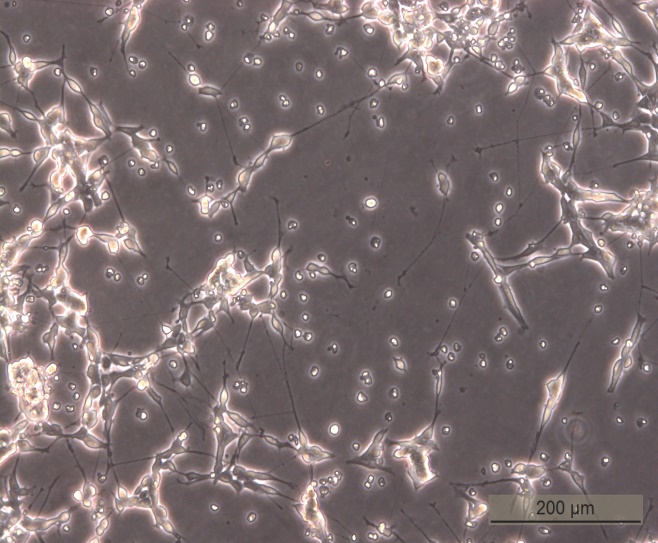

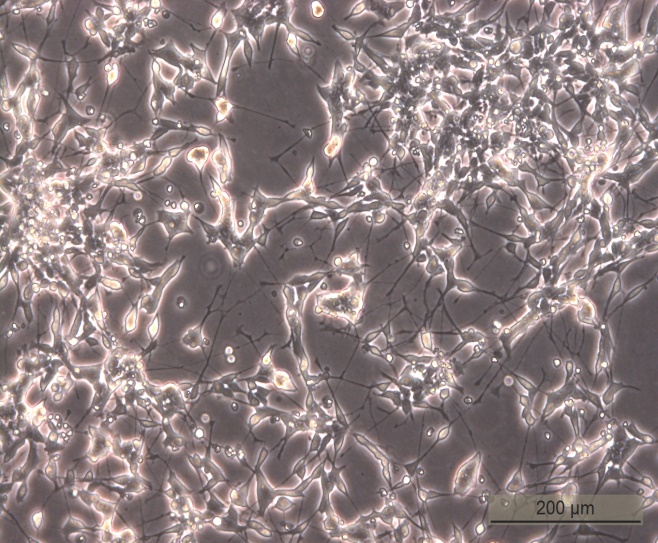

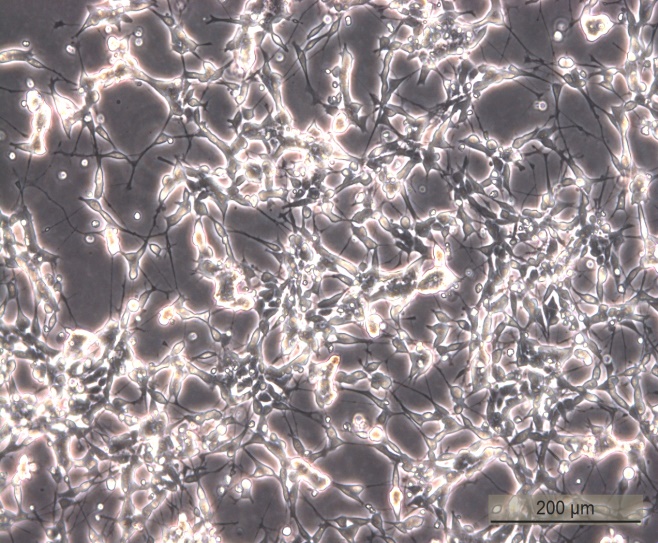


**Differentiated (Aβ only)**
